# Supplementary material for: The Impact of Inflammatory Stimuli on Xylosyltransferase-I Regulation in Primary Human Dermal Fibroblasts
Source: Biomedicines. 2022 Jun 19;10(6):1451. doi: 10.3390/biomedicines10061451 (PMC9220250; doi:10.3390/biomedicines10061451)
Supplement: Supplementary file 1 [file biomedicines-10-01451-s001.zip › biomedicines-1744484-supplementary.pdf]

Supplementary Material

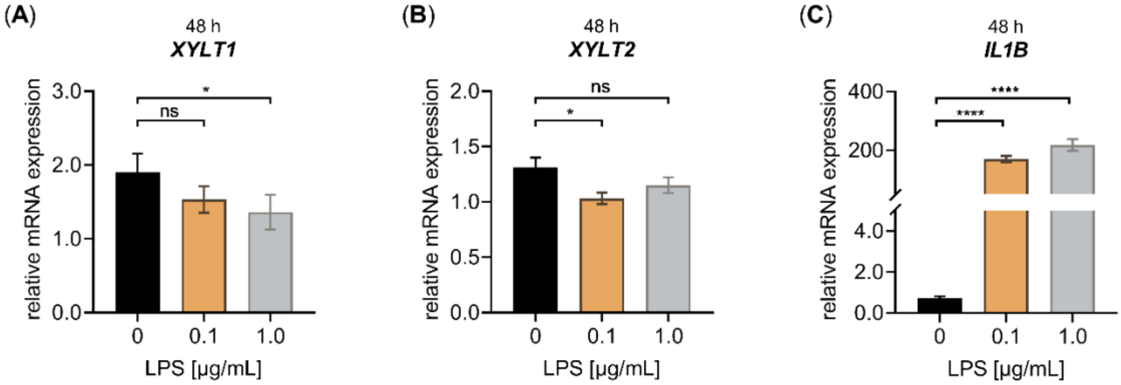

Figure S1. The effect of LPS on the relative *XYLT1*, *XYLT2* and *IL1B* mRNA expression of primary fibroblasts cultured in low-density culture conditions for 48 h. The NHDF ($n = 3$) were cultured at a cell density of 50 cells/mm^2 in growth medium supplemented with 10 % (*v*/*v*) FCS the day before the experiment. Treatment was performed with either 0 µg/mL LPS (black), 0.1 µg/mL LPS (orange) or 1.0 µg/mL LPS (grey) supplemented growth medium. The relative (**A**) *XYLT1*, (**B**) *XYLT2* and (**C**) *IL1B* mRNA-expression was analyzed after LPS treatment of NHDF for 48 h by qRT-PCR. All data are means ± SEM of three biological and three technical replicates per donor-derived primary cell culture. Mann-Whitney *U* test: ns (not significant), * $p < 0.05$ and **** $p < 0.0001$.

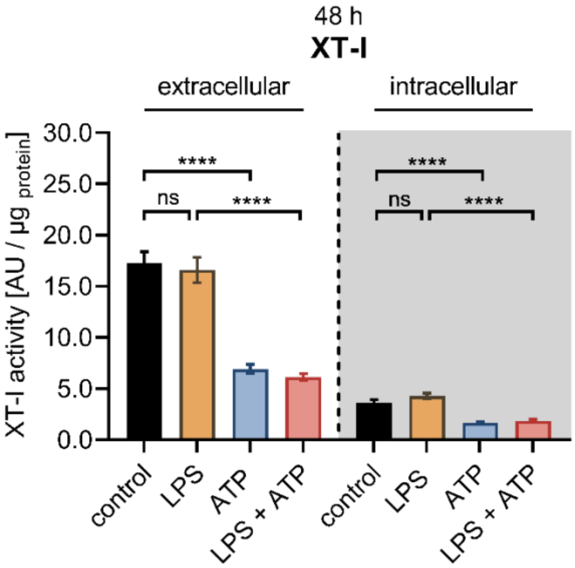

Figure S2. The cellular XT-I activity of primary fibroblasts after LPS and ATP treatment for 48 h. The NHDF ($n = 3$) were cultured at a cell density of 177 cells/mm^2 in growth medium supplemented with 10 % (*v*/*v*) FCS the day before the experiment. Cells were treated with either 0.1 µg/mL LPS (orange), 5 mM ATP (blue) or both 0.1 µg/mL LPS and 5 mM ATP (red) for 48 h. The cellular XT-I activity was determined in the cell culture supernatant (extracellular) and the corresponding cell lysates (intracellular, grey-shaded) by UPLC-ESI-MS/MS XT-I assay. The XT-I activity is expressed in AU per µg of protein. All data are means ± SEM of three biological and three technical replicates per donor-derived primary cell culture. Mann-Whitney *U* test: ns (not significant) and **** $p < 0.0001$.

Table S1. The sequences, annealing temperatures (T_A) and expected product sizes of the oligonucleotides used for qRT-PCR analysis.

gene	5′ to 3′ primer sequences	T_A / °C	product size / bp
CASP1	CAGATGCCCACCACTGAAAGA<br>GATCTCTTCACTTCCTGCCCAC	63	109
CTSB	CCACAGTGTCCCACCATCAA<br>TAGCCACCATTACAGCCGTC	63	191
IL1B	ACAGATGAAGTGCTCCTTCCA<br>GTCGGAGATTCGTAGCTGGAT	63	73
IL8	GAACTGAGAGTGATTGAGAGTGGA<br>CTCTTCAAAAACTTCTCCACAACC	63	134
